# Hypnotherapy and IBS: Time to take control of the Asian gut

**DOI:** 10.1002/jgh3.12785

**Published:** 2022-07-07

**Authors:** Kewin Tien Ho Siah, Sanjiv Mahadeva

**Affiliations:** ^1^ Division of Gastroenterology & Hepatology University Medicine Cluster, National University Hospital Singapore; ^2^ Department of Internal Medicine Yong Loo Lin School of Medicine, National University of Singapore Singapore; ^3^ Division of Gastroenterology, Department of Medicine Faculty of Medicine, University of Malaya Kuala Lumpur Malaysia

The 2014 revised definition from the American Psychological Association's Division 30 describes hypnosis as “a state of consciousness involving focused attention and reduced peripheral awareness characterized by an enhanced capacity for response to suggestion.” Hypnotherapy refers to the use of hypnosis in the treatment of a medical or psychological disorder or concern.^1^ The treatment involves a one‐to‐one 30–60 min session with a trained therapist, at weekly intervals, for 6–12 weeks.

Gut‐directed hypnotherapy (GDH) uses suggestions or metaphors to imply control and normalization of gastrointestinal function. The patient is first educated regarding basic anatomy and physiology, and how disturbance of this normal function can produce symptoms. They are then taught various skills to enable them to gain control of their gut function, often using metaphors or tactile approaches. GDH has proven benefits to patients in terms of symptom improvement and decreased medical expenditure. It is recognized as an effective psychological treatment for IBS in recent clinical guidelines.^2^ Yet despite being an effective treatment for IBS, hypnotherapy remains under‐prescribed. Why is this so?

One reason could be due to misconceptions that patients have regarding hypnotherapy. Patients commonly hold the misbelief that hypnotherapists can make them act against their will or even expose their personal secrets. In truth, hypnotherapy is used as a powerful means of teaching patients how to control their minds and bodies. Studies have shown that patients with higher hypnotic susceptibility benefit from improvement in symptom severity, health‐related quality of life, and coping patterns after hypnotherapy treatment. Some research has shown that skepticism and apprehension were often common before treatment and transformed to enthusiasm afterward.^3^

There is an additional uncertainty about the mechanism of action of hypnotherapy. It is suggested that GDH works on the brain–gut axis to improve GI symptoms in IBS patients. In patients who had undergone hypnosis therapy, brain response to distension was similar to that observed in healthy controls, suggesting that the treatment had a normalizing effect on the central processing abnormality of visceral signals in IBS.^4^ Past studies on intestinal microbiota composition and GI motility before and after hypnotherapy did not find any difference.^5^

Lack of familiarity is yet another barrier to hypnotherapy. The first hypnotherapy trial for IBS was published by Whorwell et al. in 1984. They compared the effect of psychotherapy and hypnotherapy for 30 severe refractory IBS patients. Whorwell et al. reported that hypnotherapy treatment was superior to psychotherapy but should be reserved for refractory cases as it is time‐consuming.^6^ Since then more studies have been performed to investigate the effect of hypnotherapy on IBS. A recent meta‐analysis of the effect of hypnotherapy on the treatment of IBS suggested that the data are promising and the treatment is likely to be beneficial, but concluded that more study is needed before a conclusion can be made.^7^ Furthermore, despite early suggestions that hypnotherapy should be reserved for refractory IBS patients and only performed in tertiary centers, we now know that group hypnotherapy and hypnotherapy through telemedicine or even a mobile phone application work equally well.^8^ Furthermore, the effects of GDH have been shown to last as long as the low FODMAP diet.

Lastly, cultural differences between populations may impede the uptake of hypnotherapy in IBS. Most of the evidence for hypnotherapy in IBS has been derived from studies in Caucasian patients, with a resultant lack of recommendation for this therapy among Asian patients. In this issue of JGH OPEN, Sasegbon et al. have reported the efficacy of hypnotherapy in British refractory IBS patients of Asian descent for the first time.^9^ Forty‐four British Asian (predominantly Pakistani and Indian heritage) IBS patients received hypnotherapy over 12 weeks, with significant improvement in IBS symptoms (84% achieved >50‐point reduction in IBS‐SSS) and quality of life. Although this study was conducted in routine clinical practice without a control group, the results were similar to controlled trials using the same outcome measures. Interestingly, the authors did not identify any difference in response between first‐generation British Asians and those born in the United Kingdom, nor whether the therapist was of Asian descent or not. While the current study indicates that refractory IBS patients of Asian descent are likely to benefit from hypnotherapy, it is uncertain if the same is true for IBS patients residing in Asia. It is recognized that while some cultural practices and health beliefs are retained when Asians migrate to the West, many are known to change and adopt the practices of their host countries.^10^

Hypnotherapy has a definite role to play in refractory IBS. Misconceptions and lack of awareness will need to be overcome by education and promotion. The current study by Sasegbon et al. suggests that cultural factors and health beliefs among Asian IBS patients may not be a barrier, but further studies in an Asian setting need to be conducted. However, implementing non‐pharmacological therapy for IBS patients in Asia still remains a challenge. In a recent Editorial review, Chuah et al. had highlighted that many Asian countries still utilized pure pharmacological therapy in the management of IBS and lacked resources for allied healthcare services.^11^ Furthermore, as such therapies are usually time‐intensive and require multiple visits to an institution for treatment, acceptance by patients is thought to be poor.^12^ Nevertheless, it is certainly time for Asian clinicians to acknowledge the futility of a pure pharmacological‐based approach. Asian IBS patients need to be educated accordingly and need to start “taking‐control” of their symptoms!
